# Hepatitis B virus vaccination booster does not provide additional protection in adolescents: a cross-sectional school-based study

**DOI:** 10.1186/1471-2458-14-991

**Published:** 2014-09-23

**Authors:** Yung-Chieh Chang, Jen-Hung Wang, Yu-Sheng Chen, Jun-Song Lin, Ching-Feng Cheng, Chia-Hsiang Chu

**Affiliations:** Department of Pediatrics, Hualien Tzu Chi Hospital, Buddhist Tzu Chi Medical Foundation, Hualien, Taiwan; Department of Medical Research, Hualien Tzu Chi Hospital, Buddhist Tzu Chi Medical Foundation, Hualien, Taiwan; Institute of Biomedical Sciences, Academia Sinica, Taipei, Taiwan

**Keywords:** HBV booster, Adolescents, Anamnestic response, Infant HBV vaccination

## Abstract

**Background:**

Current consensus does not support the use of a universal booster of hepatitis B virus (HBV) vaccine because there is an anamnestic response in almost all children 15 years after universal infant HBV vaccination. We aimed to provide a booster strategy among adolescents as a result of their changes in lifestyle and sexual activity.

**Methods:**

This study comprised a series of cross-sectional serological surveys of HBV markers in four age groups between 2004 and 2012. The seropositivity rates of hepatitis B surface antigen (HBsAg) and its reciprocal antibody (anti-HBs) for each age group were collected. There were two parts to this study; age-specific HBV seroepidemiology and subgroup analysis, including effects of different vaccine types, booster response for immunogenicity at 15 years of age, and longitudinal follow-up to identify possible additional protection by HBV booster.

**Results:**

Within the study period, data on serum anti-HBs and HBsAg in a total of 6950 students from four age groups were collected. The overall anti-HBs and HBsAg seropositivity rates were 44.3% and 1.2%, respectively. The anti-HBs seropositivity rate in the plasma-derived subgroup was significantly higher in both 15- and 18-year age groups. Overall response rate in the double-seronegative recipients at 15 years of age was 92.5% at 6 weeks following one recombinant HBV booster dose. Among the 24 recipients showing anti-HBs seroconversion at 6 weeks after booster, seven subjects (29.2%) had lost their anti-HBs seropositivity again within 3 years. Increased seropositivity rates and titers of anti-HBs did not provide additional protective effects among subjects comprehensively vaccinated against HBV in infancy.

**Conclusions:**

HBV booster strategy at 15 years of age was the main contributor to the unique age-related phenomenon of anti-HBs seropositivity rate and titer. No increase in HBsAg seropositivity rates within different age groups was observed. Vaccination with plasma-derived HBV vaccines in infancy provided higher anti-HBs seropositivity at 15–18 years of age. Overall booster response rate was 92.5% and indicated that intact immunogenicity persisted at least 15 years after primary HBV vaccination in infancy. Booster vaccination of HBV did not confer additional protection against HBsAg carriage in our study.

## Background

The world’s first nationwide hepatitis B virus (HBV) infant vaccination program was launched in Taiwan in July 1984, starting with newborns of highly infectious mothers and expanding to all newborns in July 1986 [1]. Prior to July 1992, infants were given four doses of plasma-derived vaccine at birth, 1, 2, and 12 months of age. After July 1992, three doses of recombinant vaccine were administered at the age of less than 1 week, 1 month, 6 months [2]. The protective cut-off level was set at ≥10 mIU/mL for antibody to hepatitis B surface antigen (anti-HBs) based on vaccine efficacy studies [3]. Over the past 20 years, the hepatitis B surface antigen (HBsAg) seropositivity rate has decreased from 9.8% in 1984 to 0.6% in 2004 among people younger than 20 years of age in Taipei, Taiwan [4–7]. Despite the success of the universal infant hepatitis B (HB) vaccination program, chronic HBV infection and hepatocellular carcinoma were not eliminated in children in Taiwan. Among the children who initially responded to the primary three-dose vaccination series, 15–50% demonstrate a low or undetectable anti-HBs level 5–15 years after primary vaccination [8]. Although perinatal hepatitis B virus transmission is still the main cause for vaccine failure [2], horizontal and breakthrough infection may also occur after waning or eventual loss of vaccine protectiveness in older children, especially with changes in lifestyle and sexual activity [9]. Currently, a booster of HB vaccination is not recommended for the general healthy population after primary immunization because of the absence of increased HBsAg seropositivity at different ages (<20 years of age), which implies that there is no increased risk of persistent HBV infection with aging [7]. Over the years, the role of the anamnestic response, indicating immune memory to HBsAg, was confirmed after anti-HBs levels had decreased to below the seroprotective level. However, a large-scale study provided evidence that an anamnestic anti-HBs response was absent in 10.1% of 15- to 18-year-old individuals in Taiwan, a country that had high endemicity of HBV [10].

In this report, we describe two parts of the present study; age-specific HBV seroepidemiology and subgroup analysis including effects of different vaccine types, immunogenicity response to booster at 15 years of age, and longitudinal follow-up to assess possible additional protection by HBV booster.

## Methods

### Vaccination program in Taiwan

The nationwide HBV infant vaccination program in Taiwan began with vaccination of newborns of highly infectious mothers in July 1984 and then expanded to all newborns in July 1986 [1]. Before July 1992, four doses (5 μg/dose) of plasma-derived vaccine were given at birth, 1, 2, and 12 months of age. After July 1992, three doses of recombinant vaccine were given at the age of less than 1 week, 1 month, and 6 months [2]. After July 1991 (birth cohort 1986), all newly enrolled elementary school first graders were required to provide their vaccination cards for mandatory check-up, and those children with incomplete vaccination records were given catch-up HBV vaccination before enrolment.

### Study population

**Figure 1:**
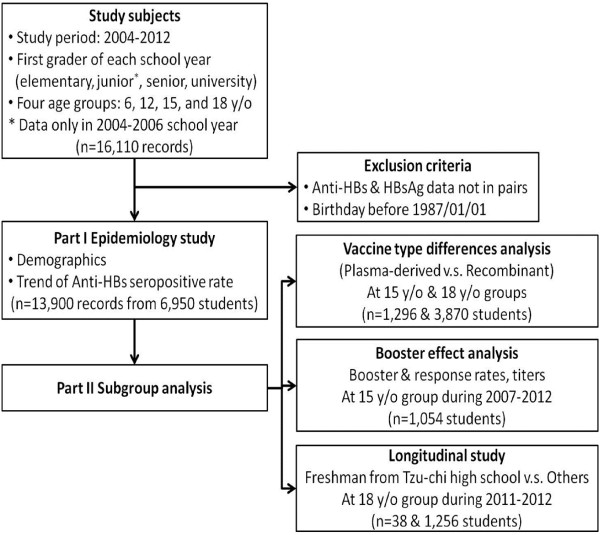
**Flow chart of study design.**

This was a retrospective cross-sectional study composed of serological surveys of HBV markers between 2004 and 2012 from newly enrolled students of the Tzu-Chi University-affiliated education system, including elementary school (birth cohort 1998–2006), junior high (birth cohort 1992–1994), senior high (birth cohort 1989–1997) schools, and university (birth cohort 1986–1994) in Eastern Taiwan. A flow chart indicating our study design is depicted in Figure 1. An approval certificate for this study was also issued from the Research Ethics Committee of Hualien Tzu Chi Hospital, Buddhist Tzu Chi Medical Foundation (REC No.: IRB101–125). A total of 16,110 records including anti-HBs or HBsAg were included in our database. We excluded subjects without records of paired HBV markers (anti-HBs/HBsAg) and those who were born before January 1st 1987, leaving a total of 6950 subjects in the epidemiological study. We hypothesized that all of our subjects received HBV vaccination within about 6 months following the introduction of the new vaccination policy at that time. Based on the statistical records of Taiwan National Immunization Information System (NIIS), the immunization coverage rates of complete HBV vaccination were 88.8–97.7% (birth cohort 1984–2013), and the complete rate of HBV vaccination among elementary school enrollments was above 99% after the 1997 birth cohort [11, 12]. Written consent forms that were provided by the Hualien County Government Education Bureau were obtained from the students’ parents or guardians upon school enrollment. The consent form informed parents/guardians that these examinations were non-intrusive with minimum risk and students or parent/guardians were free to withdraw from any examination item at any time.

### Epidemiological study

There were four groups of enrolled students: first graders in elementary school (age 6 years), junior and senior high school students (age 12 years and age 15 years) and freshmen at university (age 18 years). Blood samples were collected from each student as part of their health examination during the first semester of their enrollment. The rates of anti-HBs and HBsAg seropositivity among each group were collected and the data spanning all 9 years were analyzed. In addition to qualitative anti-HBs data, anti-HBs serum titers were available after 2007 for all school stages in our education system. The rates of anti-HBs and HBsAg seropositivity, along with other demographic characteristics such as gender, age, and median titers of anti-HBs were also compared among different age groups. In the 12-year age group, only data from the school years 2004–2006 were available in our study. Therefore, we also investigated whether there were any differences in demographic characteristics within different time spans such as 2004–2006 versus 2004–2012. There were no data for anti-HBc available within our database. Therefore, prevalence rates of natural infection could not be estimated in our study.

### Subgroup analysis

#### Comparison of HBV markers by different vaccine types

In our study subjects, we noted some were vaccinated with a plasma-derived HBV vaccine and others received recombinant HBV vaccine in infancy, based on their birth cohort. Subjects born before July 1992 received four doses of plasma-derived vaccine (Hevac B; Pasteur-Merieux, or its equivalent derivative); others, who were born after July 1992, received recombinant HBV vaccines (5 μg/dose of Recombivax [Merck] or 20 μg/dose of Engerix [GSK]) [1]. Therefore, we selected subjects by their birth cohort, grouping them as either plasma-derived or recombinant vaccine recipients. In the 15-year age group, subjects from birth cohorts 1989–1991 and 1993–1997 were grouped as plasma-derived and recombinant groups, respectively. In the 18-year age group, subjects from birth cohorts 1987–1991 and 1993–1994 were defined as plasma-derived and recombinant groups, accordingly. We compared the seroprevalence of anti-HBs and HBsAg following administration of the different vaccine types in these two age groups.

#### Immunogenicity response in booster recipients at 15 years of age

In our database, subjects in birth cohort 1992–1997 demonstrating double seronegativity for anti-HBs and HBsAg in the 15-year age group were recommended to receive one booster dose of recombinant HBV vaccine (20 μg/dose). One booster dose was to be administered in their school clinic on the same day under the supervision of the family physician. Post-booster blood sampling for anti-HBs titers were also performed on the same day in the school clinic an average of 6 weeks (range 35–50 days) after their booster date. The response rate to one booster dose of HBV vaccine was defined as the proportion of booster recipients whose post-booster titer was ≥10 mIU/mL. The overall and annual anti-HBs seropositivity rates after one booster dose of HBV vaccine were also calculated. Anti-HBs seropositivity rates after one booster dose were defined by the following equation:


This equation was modified from the formula by Wu et al. [13]. In their study, the overall anti-HBs seropositive rate, designated as PR_T_, after a booster dose of HB vaccine was approximated by the following formula:


where PR_1_ is the anti-HBs seropositive rate the before booster, and PR_2_ is the response rate in the booster recipients.

All of the seronegative subjects had received a booster dose in the Wu et al. study [13]. Therefore, no booster rate was needed in their formula. In our study, the booster rate was defined as the percentage of seronegative subjects receiving one booster dose. The response rate was defined as the proportion of those booster recipients whose post-booster anti-HBs titer was ≥10 mIU/mL.

#### Longitudinal study

In our database of the university-affiliated education system, we found by birth cohort tracing, that some of our subjects studied in our senior high school and then consequently in our university. We decided to follow the subjects of birth cohort 1993–1994 in the 18-year age group who had received the recombinant HBV vaccine in infancy. In this way, the effects of different vaccine type were eliminated. If the subjects previously studied in our senior high school (15-year age group, birth cohort 1993–1994), they were grouped as “the same school”. Their serum data of both anti-HBs and HBsAg were analyzed longitudinally to trace the pattern of seroconversion and post-booster change in this 3-year interval. The other subjects of the 18-year age group from the 1993–1994 birth cohort were used as the comparison group, which was labeled as “the others”. No records of booster history in “the others” group was available, therefore, an uncertain proportion of subjects in this group may have received a booster dose after their HBV vaccination in infancy. We hypothesize that median value of anti-HBs before HBV booster among subjects in birth cohort 1993–1994 in the 15-year age group could serve as the baseline median value in the 18-year age group. The actual median titers of anti-HBs in the 18-year age group would be further decayed if a booster dose was not given or natural exposure did not occur. In addition to the seropositivity rate of anti-HBs and HBsAg, median values of anti-HBs titers at 18 years of age were compared among two groups, “the same school” versus “the others”, to identify if booster effects were present in our comparison group. We aimed to identify any additional protective effect by increasing the levels of anti-HBs titers by comparing the HBsAg seropositivity rate between these two groups.

### Serologic testing

All quantification of seromarkers of HBV infection was performed by enzyme immunoassay (data prior to 2011/08/31, VITROS ECiQ Immunodiagnostics system, Ortho Clinical Diagnostics; data after 2011/09/01, Abbott Laboratories, North Chicago, IL, USA). Reference concentrations from the WHO were used as criteria values and the protective level of anti-HBs was defined as ≥10 mIU/mL [3]. Subjects who were positive for HBsAg were assumed to be hepatitis B carriers [14].

### Statistical analysis

A Chi-square test was performed to identify the differences in seropositivity rates, including anti-HBs and HBsAg, median titers of anti-HBs, between different ages, genders, and other subgroups. Statistically significant differences were defined as p < 0.05. All of the statistical analyses were performed using SPSS software (version 17.0; SPSS Inc., Chicago, IL, USA).

## Results

### Part I: epidemiological study

**Figure 2:**
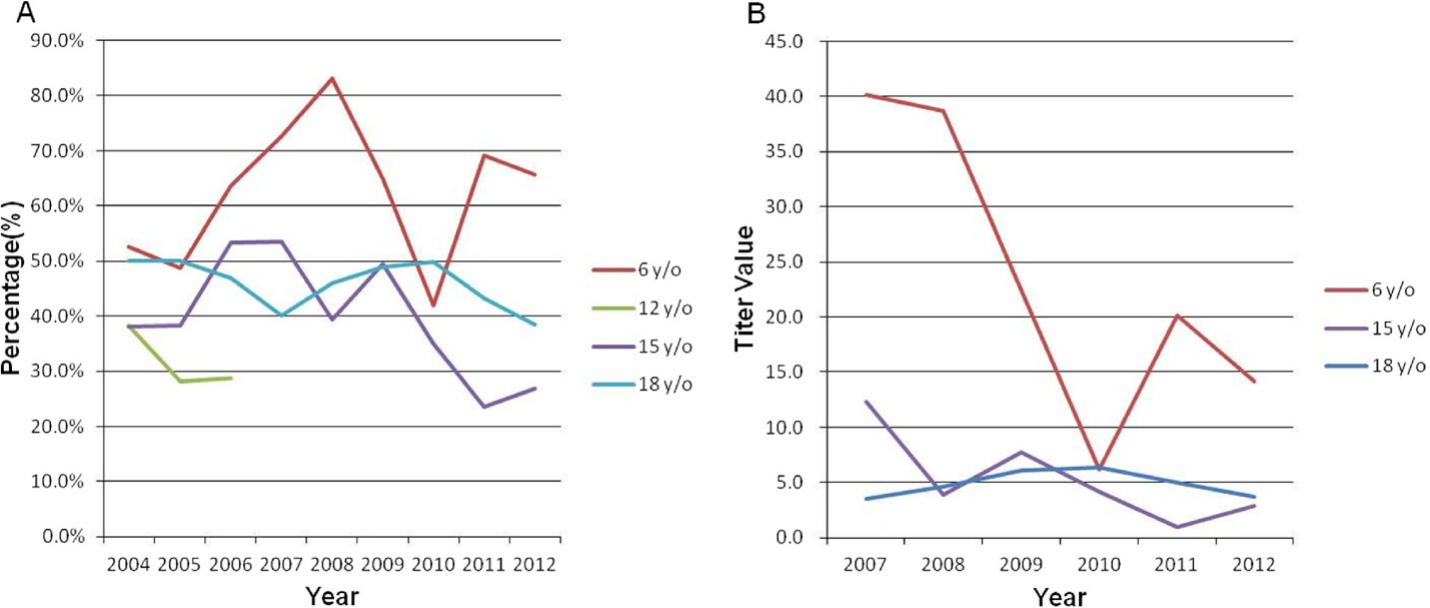
**The trend of anti-HBs seropositivity rate and median titers by different age group and time frame. (A)** The trend of anti-HBs seropositive rate by age (2004-2012). **(B)** The trend of median titer value of anti-HBs by age (2007-2012).

The study subjects of this epidemiological survey consisted of 6950 individuals (2901 were male and 4049 were female), all of whom were born after 1987, within the national infant HBV vaccination era of Taiwan. There were 524, 450, 1464, and 4512 individuals in the 6-, 12-, 15-, and 18-year age groups, respectively. The overall seropositivity rate of anti-HBs and HBsAg was 44.3% and 1.2%, respectively, in our study subjects. Figure 2 shows the trend of anti-HBs seropositivity rate and median titers by different age group and time frame. Table 1 shows a statistically significant difference between age and the seropositivity rate of anti-HBs, with a declining rate from 6 years to 12 years of age, which then rises through 18 years of age in our study (p < 0.001). However, the age-specific pattern of HBsAg seropositivity did not show any statistical significance (p = 0.154) with aging. The median titers of anti-HBs also showed statistical significance with a pattern similar to that observed for anti-HBs seropositivity. To assess the possible effects of a shortage of data within the 12-year age group from 2007 to 2012, we analyzed the seropositive rates of anti-HBs and HBsAg in all four age groups within the same interval (2004–2006). A similar age-related pattern of anti-HBs seropositivity was noted with a statistically significant difference among all individuals (p < 0.001). We further pooled the two younger and two older age groups (6–12 vs 15–18 years) for comparison, and the results showed a similar pattern to the four age group comparison. As compared with seroprevalence data of HBV markers by genders of all subjects, female individuals had a significantly higher anti-HBs seropositivity rate (p < 0.009) than male individuals, but this was not mirrored by HBsAg (p = 0.439).

**Table 1:** **Demographics data and seroprevalence of hepatitis B virus (HBV) markers and titers**

Age group	6 y/o	12 y/o	15 y/o	18 y/o	6-12 y/o	15-18 y/o
Item	(n = 524)	(n = 450)	(n = 1464)	(n = 4512)	(n = 974)	(n = 5976)
Gender						
Male, n (%)	299(57.1)	205(45.6)	740(50.5)	1657(36.7)	504(51.7)	2397(40.1)
Female, n (%)	225(42.9)	245(54.4)	724(49.5)	2855(63.3)	470(48.3)	3579(59.9)
Age (Mean ± SD, yr)	6.5 ± 0.3	12.5 ± 0.3	15.5 ± 0.5	18.7 ± 0.7	9.3 ± 3.0	17.9 ± 1.5
Anti-HBs (+), n	332	142	574	2033	474	2607
(%, 95% CI)	(63.4, 59.3-67.5)	(31.6, 27.3-35.9)	(39.2, 36.7-41.7)	(45.1, 43.6-46.6)	(48.7, 45.6-51.8)	(43.6, 42.3-44.9)
Anti-HBs (−), n	192	308	890	2479	500	3369
(%, 95% CI)	(36.6, 32.5-40.7)	(68.4, 64.1-72.7)	(60.8, 58.3-63.3)	(54.9, 53.4-56.4)	(51.3, 48.2-54.4)	(56.4, 55.1-57.7)
P-value	<0.001*				0.003*	
Anti-HBs median titer	21.2	NA	3.9	4.8	21.2	4.6
P-value	<0.001*				<0.001*	
HBsAg (+), n	1	7	16	56	8	72
(%, 95% CI)	(0.2, 0.0-0.6)	(1.6, 0.4-2.8)	(1.1, 0.6-1.6)	(1.2, 0.9-1.5)	(0.8, 0.2-1.4)	(1.2, 0.9-1.5)
HBsAg (−), n	523	443	1448	4456	966	5904
(%, 95% CI)	(99.8, 99.4-100.0)	(98.4, 97.2-99.6)	(98.9, 98.4-99.4)	(98.8, 98.5-99.1)	(99.2, 98.6-99.8)	(98.8, 98.5-99.1)
P-value	0.154				0.336	

Note: Anti-HBs, antibody to HBV surface antigen; HBsAg, HBV surface antigen; y/o, year old; NA, not available; 95% CI, 95% confidence interval; Data are presented as or n and percentage.

*p-value < 0.05 was considered statistically significant after test.

### Part II: subgroup assessment

#### Comparison of HBV markers by different vaccine types

In this part of our study, we selected the subjects by their birth cohort, grouping as plasma-derived and recombinant groups in two age groups. We compared the seroprevalence of anti-HBs and HBsAg within the different vaccine types in these two age groups. Table 2 shows that the seropositivity rate of anti-HBs in the plasma-derived subgroup was significantly higher in both the 15- and 18-year age groups (p < 0.004 and 0.003, respectively). The seropositivity rate of HBsAg in the plasma-derived subgroup was also significantly higher in the 18-year age group (p = 0.049), but not in the 15-year age group (p = 0.129).

**Table 2:** **Comparison of seroprevalence of hepatitis B virus (HBV) markers by different age and vaccine type received in infancy**

Age group	Vaccine type	Plasma-derived	Recombinant	Total	P-value
	Items				
15 y/o	N [birth cohort]	410 [1989–91]	886 [1993–97]	1296	
	anti-HBs (+), n	177	307	484	0.004*
	(%, 95% CI)	(43.2, 38.4-48.0)	(34.7, 31.6-37.8)	(37.3, 34.3-39.9)	
	anti-HBs (−), n	233	579	812	
	(%, 95% CI)	(56.8, 52.0-61.6)	(65.3, 62.2-68.4)	(62.7, 60.1-65.3)	
	HBsAg (+), n	7	6	13	0.129
	(%, 95% CI)	(1.7, 0.4-3.0)	(0.7, 0.2-1.2)	(1.0, 0.5-1.5)	
	HBsAg (−), n	403	880	1283	
	(%, 95% CI)	(98.3, 97.0-99.6)	(99.3, 98.8-99.8)	(99.0, 98.5-99.5)	
18 y/o	N [birth cohort]	2575 [1987–91]	1295 [1993–94]	3870	
	anti-HBs (+), n	1184	529	1713	0.003*
	(%, 95% CI)	(46.0, 44.1-47.9)	(40.8, 38.1-43.5)	(44.3, 42.7-45.9)	
	anti-HBs (−), n	1391	766	2157	
	(%, 95% CI)	(54.0, 52.1-55.9)	(59.2, 56.5-61.9)	(55.7, 54.1-57.3)	
	HBsAg (+), n	40	10	50	0.049*
	(%, 95% CI)	(1.6, 1.1-2.1)	(0.8, 0.3-1.3)	(1.3, 0.9-1.7)	
	HBsAg (−), n	2535	1285	3820	
	(%, 95% CI)	(98.4, 97.9-98.9)	(99.2, 98.7-99.7)	(98.7, 98.3-99.1)	

Note: anti-HBs, antibody to HBV surface antigen; HBsAg, HBV surface antigen; y/o, year old; Data are presented as or n and percentage.

*p-value < 0.05 was considered statistically significant after test.

#### Immunogenicity response in booster recipients in the 15-year age group

Among 1054 subjects of birth cohort 1992–1997 in the 15-year age group, 657 subjects (62.3%) demonstrating seronegativity for both anti-HBs and HBsAg were further recommended to receive one booster dose of recombinant HBV vaccine (20 μg/dose). Table 3 shows that 570 (86.8%) of these 657 double seronegative subjects received one booster dose and the overall response rate among booster recipients with post-booster titer ≥10mIU/mL was 92.5% (529/570). The overall anti-HBs seropositivity rate before one booster dose was 37.7% (397/1054), while the overall anti-HBs seropositivity rate after one booster dose was 87.7%. The annual post-booster rate had a range of 83.4–93.6% in the 1992–1997 birth cohort. The median titers of anti-HBs before and after booster dose were 1.1 mIU/mL and 545.5 mIU/mL, respectively.

**Table 3:** **Booster response rate and change of overall anti-HBs seropositive rate before and after one booster dose**

Year	Seroprevalence, n (%)		Subjects, n (%)		Post-booster	Anti-HBs titer (median)	
[birth cohort]	Anti-HBs (+)	Anti-HBs (−)	Booster rate	Response rate	Anti-HBs (+)	Before	After
2007 [1992]	90 (53.6)	78 (46.4)	66 (84.6)	55 (83.3)	86.3%	2.1 ± 2.3	399.4 ± 394.0
						(P50 = 1.4)	(P50 = 288.5)
2008 [1993]	67 (39.4)	103 (60.6)	90 (87.4)	75 (83.3)	83.5%	1.7 ± 2.1	480.7 ± 423.4
						(P50 = 0.7)	(P50 = 365.5)
2009 [1994]	85 (49.4)	87 (50.6)	76 (87.4)	77 (100.0)	93.6%	1.5 ± 2.2	678.4 ± 385.5
						(P50 = 0.3)	(P50 = 975.0)
2010 [1995]	61 (35.1)	113 (64.9)	96 (85.0)	85 (87.6)	83.4%	2.7 ± 2.0	540.2 ± 421.2
						(P50 = 2.3)	(P50 = 556.5)
2011 [1996]	40 (23.7)	129 (76.3)	112 (86.8)	111 (99.1)	89.3%	1.2 ± 2.0	589.3 ± 377.2
						(P50 = 0.2)	(P50 = 555.4)
2012 [1997]	54 (26.9)	147 (73.1)	130 (88.4)	126 (96.9)	89.5%	2.6 ± 2.8	579.2 ± 387.7
						(P50 = 1.7)	(P50 = 573.7)
Total	397 (37.7)	657 (62.3)	570 (86.8)	529 (92.5)	87.7%	2.0 ± 2.3	551.5 ± 403.6
						(P50 = 1.1)	(P50 = 545.5)

#### Longitudinal study

**Figure 3:**
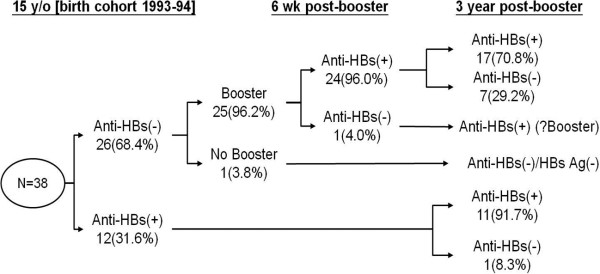
**Change of hepatitis B virus (HBV) seromarkers follow up for 3-year after HBV booster.**

Among 1294 subjects in the 18-year age group of the 1993–1994 birth cohort, only 38 (2.9%) subjects had studied in our senior high school (15-year age group, birth cohort 1993–1994), followed by attendance at our university, grouped as “the same school”. The other 1256 (97.1%) subjects of the 18-year age group within the same birth cohort were labeled as “the others”, meaning they graduated from another senior high school with unclear booster status. Figure 3 shows that the anti-HBs seropositivity rate of these 38 subjects in “the same school” group at 15 years of age was 31.6% (12/38), while 68.4% (26/38) of them demonstrated double seronegativity of anti-HBs/HBsAg at this age. Among 26 double seronegative subjects, 96.2% (25/26) received one booster dose of HBV vaccine at age 15 years. The response rate at 6 weeks after the HBV booster among these 25 recipients was 96.0% (24/25). The overall anti-HBs seropositivity rate for all “the same school” subjects at 6 weeks and 3 years after one booster dose was 94.8% and 76.3%, respectively. Among the 24 recipients showing anti-HBs seroconversion at 6 weeks after booster, seven subjects (29.2%) had lost their anti-HBs seropositivity again within 3 years. The lone double seronegative subject in “the same school” group who did not receive a booster (1/26) retained her double seronegative status for the duration of the 3-year follow-up interval. For those 12 subjects with anti-HBs seropositive status at 15 years of age, 91.7% (11/12) of them still retained anti-HBs seropositivity after 3 years.

We also compared the 38 subjects of “the same school” group with 1256 subjects of “the others” group from the birth cohort 1993–1994 in the 18-year age group to determine if higher rates of anti-HBs seropositivity did indeed provide better protection against HBV infection. Table 4 shows that the seropositivity rates of anti-HBs among “the same school” group versus “the others” group were 76.3% and 39.7%, respectively (p < 0.001). Median titers of anti-HBs were also shown to be significantly different between these two groups (i.e., 24.7 vs 4.2 mIU/mL, in “the same school” and “the others”, respectively, p <0.001). There were no new cases of HBsAg seropositivity in “the same school” group, and the seroprevalence rate of HBsAg was 0.8% in “the others” group.

**Table 4:** **Comparison of seroprevalence of HBsAg and anti-HBs in “the same school” and “the others” groups**

Item	18-year age group (birth cohort 1993–1994)		
	The same school (n = 38)	The others (n = 1256)	P-value
Anti-HBs (+), n (%, 95% CI)	29 (76.3, 62.8-89.8)	499 (39.7, 37.0-42.4)	<0.001*
Anti-HBs (−), n (%, 95% CI)	9 (23.7, 10.2-37.2)	757 (60.3, 57.6-63.0)	
Anti-HBs titer, mean ± SD (median)	157.6 ± 281.3 (P50 = 24.7)	58.8 ± 145.9 (P50 = 4.2)	<0.001*
HBsAg (+), n (%)	0 (0.0)	10 (0.8%)	0.581
HBsAg (−), n (%)	38 (100.0)	1246 (99.2%)	

Data are presented as n and percentage or mean ± standard deviation (median).

*p-value < 0.05 was considered statistically significant after test.

## Discussion

In previous studies conducted in Taiwan, the seropositivity rate of anti-HBs declined from 99% at 1 year of age to 83% at 5 years of age [15] and further dropped to 71.1% at 7 years of age, 37.4% at 12 years of age and 37% at 15–17 years of age [16]. The HBsAg seropositivity rate in children less than 12 years of age decreased from 9.8% in 1984 to 1.3% in 1994. The seropositivity rate of anti-HBc also decreased from 26% in 1984 and 15% in 1989 to 4% in 1994 [1]. In a 2004 survey, Ni et al. showed that the anti-HBc seropositivity rate was low (1%) in children less than 15 years of age [7]. In 2004, the seropositivity rates for HBsAg, anti-HBs, and anti-HBc were 1.2%, 50.5%, and 3.7%, respectively, in those born after implementation of the vaccination program (age <20 years) [7].

In our study, the overall seropositivity rates of HBsAg and anti-HBs were 1.2% and 43.6%, respectively, in those individuals less than 18 years of age. In Table 1, we noted a phenomenon in which the seropositivity rate of anti-HBs decayed from 63.4% (age 6 years) to 31.6% (age 12 years) and then increased from 12 to 18 years of age. To the best of our knowledge, there is no research reporting a phenomenon similar to the data presented in Figure 2. Reactivation effects (HBV vaccine booster), natural boosters (boosting of the anti-HBs titer after a natural exposure), and breakthrough infections (having HBV infection despite receiving three or more doses of HBV vaccine) could all contribute to the pattern of increase in anti-HBs from 12 to 18 years of age as observed in this study. Tracing our subjects by their birth cohort (1992–1994) in the 12-year age group, we found 110 subjects (110/510, 21.6% of total enrolled students of senior high school in the corresponding period) had also enrolled in our senior high school at 15 years of age. Of subjects with seronegative anti-HBs titers at 12 years of age, 78.8% (63/80) were found to be seropositive at age 15 years (data not shown). We were unable to trace their booster records individually, although such seroconversion was highly indicative of booster effects, which may be a factor that interfered with the anti-HBs seropositivity rate in this birth cohort in the 15-year age group. Similarly, booster effects might also contribute to the elevated seropositivity rate and median titers of anti-HBs in the 18-year age group with less amplitude of influence if compared with those values in the 15-year age group.

In previous studies, the anti-HBc positivity rate of elementary school first graders within the 3-year follow-up period (2005–2007, birth cohort 1999) was between 1 and 2% in the North, Central, and South of Taiwan but was higher (3.5–4.8%) in Eastern Taiwan [17]. Another survey conducted in 2004 among children younger than 18 years of age in Taiwan showed breakthrough HBV infection was about 1% in children sampled (136/13765) [7]. Therefore, the unique phenomenon of increasing anti-HBs seropositivity rate after 12 years of age was likely because of booster reactivation effects. Poovorawan et al. reported that breakthrough infections during the first decade after primary vaccination in infancy were only observed in children born to high-risk families (maternal seropositive for both HBsAg and HBeAg). In the second decade after primary vaccination, breakthrough infections were detected in 12.9% of individuals, possibly reflecting increased exposure outside the home, linked to high-risk adolescent behaviors [18].

The highest rate of HBsAg seropositivity was 1.6% in the 12-year age group of this study, but no statistical significance (p = 0.154) was observed in the pattern of anti-HBs seroprevalence by age. All students in the 12-year age group were born between 1992 and 1994 and their HBsAg seropositivity rate was consistent with other studies within the same time frame [17]. Natural HBV infections (positive for anti-HBc) and HBV carriers (seropositive HBsAg) were found in 4.1% and 1.6% of individuals, respectively, at 15–17 years of age in a post-1986 cohort in Taiwan [10].

Based on data from previous studies, different types of HB vaccines, doses, and brands as well as the timing of primary vaccination can all influence the persistence of anti-HBs titers [19]. In Taiwan, neonates born before July 1992 received four doses of plasma-derived vaccine (Hevac B; Pasteur-Merieux, or its equivalent derivative); neonates born after July 1992 received recombinant HBV vaccines (5 μg/dose of Recombivax [Merck] or 20 μg/dose of Engerix [GSK]) instead. In Table 2, we demonstrate that individuals in the plasma-derived subgroup had a higher proportion of positivity for anti-HBs than did those of the recombinant subgroup in both the 15- and 18-year age groups (43.2% vs 34.7% at 15 years, p < 0.004; 46.0% vs 40.8% at 18 years, p < 0.003). Our finding is consistent with several other studies [20, 21]. The seropositivity rate of HBsAg in the plasma-derived subgroup is significantly higher than that of the recombinant subgroup in the 18-year age group (p = 0.049) alone, but not in the 15-year age group (p = 0.131). In our subsequent study, only the subjects who received recombinant HBV vaccine were pooled together for a longitudinal study to avoid the interference of different vaccine type and dosage given in infancy.

According to the birth cohort (1987–2003) analysis for long-term immunity following infant HBV vaccination, no increase of the seropositivity rates of HBsAg and anti-HBc was noted after 17 years of age [7]. In contrast, 15–50% of the children who initially responded to the three-dose series of HBV vaccination had low or undetectable anti-HBs levels 5–15 years after primary vaccination [8]. Wu et al. reported that the overall anti-HBs seropositivity rate after a booster dose of HB vaccine was estimated to be 84.3% among 1974 students from senior high schools with negative titers of both anti-HBs and HBsAg before the HB vaccine booster [13]. Long-term protection studies indicated that immunological memory usually persisted even if anti-HBs levels fell below the protective threshold (10 mIU/mL) [20, 21]. However, without protective levels of anti-HBs, memory cells alone are probably unable to protect against acute infection [22]. In our subjects within the 15-year age group from the birth cohort 1992–1997 presented in Table 3, booster response rate was 92.5% (529/570) after one booster dose of recombinant HBV vaccine (20 μg/dose). Anti-HBs seropositivity rate before one booster dose was 37.7% (397/1054) and became 87.7% afterwards. The median titers of anti-HBs before and after booster dose were 1.1 mIU/mL and 545.5 mIU/mL, respectively. All the subjects received recombinant HBV vaccine in their neonatal period, except the individuals from the 1992 birth cohort, who received plasma-derived HBV vaccine if they were born before July 1992. Lu et al. showed the booster response rate to one dose of recombinant HBV vaccine was 71% in 15- to 17-year-old subjects who received plasma-derived HBV vaccine during neonatal immunization [10]. All the subjects from the Wu et al. [13] study were born between July 1987 and July 1991 and thus belonged to the plasma-derived HBV vaccine era in Taiwan. Therefore, the booster response pointed to the recipients who received the plasma-derived vaccine in infancy.

In Figure 3, we selected and followed 38 subjects who had graduated from our senior high school and consequently studied in our university. We monitored the changes in the seropositivity rates of anti-HBs and HBsAg within a time frame of 3 years. We found that the initial booster response rate at 6 weeks after one booster dose of HBV vaccine was as high as 96% (24/25) in our 15-year-old subjects. Surprisingly, seven subjects (29.2%, 7/24 booster responders) who became seropositive after their booster dose lost their anti-HBs seropositivity again within 3 years after booster. The overall anti-HBs seropositivity rate for all “the same school” subjects at 6 weeks and 3 years after one booster dose was 94.8% and 76.3%, respectively. For the comparison group labeled as “the others”, subjects from the same birth cohort in the 18-year age group, the seropositivity rates of anti-HBs and HBsAg were 39.7% (499/1256) and 0.8% (10/1256), respectively. The median titer of subjects in “the others” group was 4.2 mIU/mL (24.7 mIU/mL in “the same school”). During the 3-year follow-up period, no new HBV carriers were detected in “the same school” group, which included students given a booster dose if their anti-HBs titers were seronegative. However, there was no statistical difference (p = 0.581) in HBV carrier rate between “the same school” subgroup and “the others” subgroup (0% vs 0.8%, respectively). An HBsAg seropositivity rate of 0.8% at 18 years of age in our study group was comparable to the seroprevalence of the same age group in other studies [7]. In our study, booster vaccination did not confer additional protection against HBsAg carriage. High booster response rate (96%) at 6 weeks after one booster dose at 15 years of age also indicated intact long-term protection of HBV infection by immunological memory, as a result of anamnestic response after primary infant HBV vaccination. Immunity against HBV provided protection against infection as well as against disease. Protection against infection is associated with antibody persistence, which is directly related to the peak production of anti-HBs after primary vaccination. Protection against disease (i.e., acute hepatitis, prolonged viraemia, carriership, and chronic infection) is associated with immune memory that persists beyond the time at which anti-HBs disappears [9]. Currently, Middleman et al. show several variables independently associated with higher geometric mean titer response to a challenge dose of vaccine included a higher baseline anti-HBs titer, older age at first dose of primary series (≥4 weeks after birth), higher test dosage, and non-white race [23]. In contrast to the high endemicity of HBV in Taiwan, Middleman et al. collected these new data regarding duration of protection in the setting of low hepatitis B endemicity in the United States, with a likely absence of natural boosting. The response to the challenge dose of HB vaccine was remarkably good, as high as 92% [23], which is similar to our booster response rate after one booster dose of HBV vaccine at 15 years of age.

Taking Taiwan as an example of an HBV endemic area, the seropositivity rates of HBsAg and HBeAg for pregnant women were still 10.3% and 2.3% in 2009 (data from Taiwan Centre for Disease Control, Department of Health). Additionally, the mean age of mothers giving birth in 2007–2009 was 29.45 years of age, which meant the majority were born after the introduction of infant HBV vaccination. Horizontal and breakthrough infection could also occur after waning or eventual loss of the vaccine protectiveness in older children, especially with changes in lifestyle and sexual activity [9]. A concern exists about sexual exposure to HBsAg carriers in hyperendemia areas such as Taiwan when vaccinated children become adolescents and young adults. Ni et al. showed no increase in seropositivity rates of either HBsAg or anti-HBc when vaccinated individuals progressed to 17 years of age [7] and the rate of chronicity declined as the age of infection increased: 25% in infected preschool children and 3–10% in adolescents and young adults [24]. Although universal booster dose may not be necessary up to 20 years after the primary vaccination because HBsAg and anti-HBc seropositivity did not increase [7], the methods for the prevention of horizontal transmission, such as avoidance of skin tattooing, use of disposable needles, and condom use in sexual contact still need continuous implementation in the adolescent group [25]. Based on our data, the current guidelines from Taiwan Advisory Committee on Immunization Practice (ACIP) appear to be adequate, and states that individuals may receive a booster dose if they have negative anti-HBs antibodies and are in high-risk groups (i.e., hemodialytic, organ transplant, and immunocompromised patients; intravenous drug users; participants in high-risk sexual activity; or health care workers).

The major limitations of our study were as follows. First, the retrospective cross-sectional study design and records analysis was conducted without reviewing any vaccination records for all subjects. Second, quantitative data of anti-HBs titers were available for all four age groups in the period of 2007–2012 but anti-HBc was not routinely screened for during entrant health-screening examination owing to a decreased seropositivity rate of this marker [26, 27] and a high proportion of HBV-DNA negativity in anti-HBc positive subjects [28]. As a result, the seropositivity rates of natural infection could not be estimated in our study. Third, despite achievement of high HBV vaccine coverage rates from 88.8% to 96.9% (from 1984 to 2010) according to data from the Centre for Disease Control in Taiwan [11, 12], there was a possible bias in primary versus booster doses in our study subjects. The proportion of booster rate in “the others” subgroup analysis could not be identified and therefore we could only state possible booster dose effects according to the level of anti-HBs titers when comparing these data to the value of anti-HBs in subjects from the same birth cohort.

## Conclusions

HBV booster strategy at 15 years of age was the main contributor to the unique age-related phenomenon of anti-HBs seropositive rate and titers. No increase in HBsAg seropositivity rates within different age groups (p = 0.154) was observed. Female individuals demonstrated significantly higher anti-HBs seropositivity compared with male individuals (p < 0.015). Vaccination with plasma-derived HBV vaccines in infancy provided a higher rate of anti-HBs seropositivity at 15–18 years of age in our study than the recombinant vaccine. The overall response rate at 6 weeks after one HBV booster dose was 92.5% in subjects demonstrating double seronegativity for anti-HBs and HBsAg, which indicated that intact immunogenicity persisted at least 15 years after primary infant HBV vaccination. Booster vaccination of HBV did not confer additional protection against HBsAg carriage in our study. Therefore, based on our data, we conclude that the current booster strategy implemented by Taiwan ACIP, which states that individuals may receive a booster dose if they have negative anti-HBs antibodies and are in high-risk groups (i.e., hemodialytic, organ transplant, and immunocompromised patients; intravenous drug users; participants in high-risk sexual activity; or health care workers), remains adequate.
